# Reactivation of *P53* Antiproliferative and Pro-Apoptotic Pathways by Resveratrol in Mutant *P53* Cancer Cell Lines

**DOI:** 10.3390/ijms27104481

**Published:** 2026-05-16

**Authors:** Andrea Acosta-Dent, Enrique García-Villa, Sandra Cotino-Nájera, Solangy Lizcano-Meneses, Francisco Alejandro Lagunas-Rangel, Efraín Garrido-Guerrero, José Díaz-Chávez, Patricio Gariglio

**Affiliations:** 1 Departamento de Genética y Biología Molecular, Centro de Investigación y de Estudios Avanzados (CINVESTAV-IPN), Av. IPN No. 2508, Gustavo A. Madero, Mexico City 07360, Mexico; 2 Subdirección de Investigación Básica, Instituto Nacional de Cancerología, Av. San Fernando No. 22, Sección XVI, Tlalpan, Mexico City 14080, Mexico; 3 Tecnológico de Monterrey, Escuela de Medicina y Ciencias de la Salud, Calle del Puente #222, Col. San Bartolo el Chico, Tlalpan, Mexico City 14380, Mexico

**Keywords:** cancer, mutant *p53*, reactivation, chemosensibilization, resveratrol

## Abstract

Cancer is the second leading cause of death worldwide. Mutations in the *TP53* gene lead to a loss of tumor suppressor function and an oncogenic gain of function for the protein, resulting in a more invasive, metastatic, and chemoresistant phenotype. Diverse structural studies have demonstrated that mutant *p53* core domain unfolding is not irreversible. Thus, reactivation toward its wild-type-like conformation or inactivation of its mutant *p53* capacities may restore the expression of genes in its tumor suppressor pathways, resulting in enhanced responses to current therapies. Resveratrol (3,4′,5-trihydroxy-trans-stilbene) is a phytoalexin naturally found in more than 70 plant species that has widely proven antiproliferative and pro-apoptotic properties, as well as a capacity to reverse multidrug resistance in various cancer types. Interestingly, it has recently been demonstrated that resveratrol directly interacts with the *p53* core domain and reduces mutant *p53* aberrant aggregation. In this context, our study aims to elucidate whether resveratrol may induce antiproliferative and pro-apoptotic pathways regardless of a mutant background. We observed that resveratrol has an antiproliferative effect in cancer cells, independent of *p53* status, and leads to apoptosis after 48 h of treatment. Resveratrol also induces the expression of *p53* tumor suppressor target genes, which are involved in cell cycle arrest and apoptosis. Even though the previous effects are more significant in cells expressing wild-type *p53*, resveratrol drastically sensitizes all cancer cell lines, regardless of *p53* status, to cisplatin treatment, making it a promising enhancer compound to overcome chemoresistance associated with *p53*.

## 1. Introduction

Cancer is the second leading cause of death worldwide [1]. Tumorigenesis is driven by the accumulation of multiple mutations and epigenetic alterations in oncogenes and tumor suppressor genes [2,3]. The *TP53* gene encodes the tumor suppressor protein p53, which plays a critical role in preventing carcinogenesis. Upon activation by diverse stresses, including DNA damage, oncogene activation, oxidative stress, hypoxia, and nutrient deprivation, *p53* acts as a transcription factor that triggers a variety of antiproliferative programs involved in multiple cellular responses, such as cell cycle arrest, DNA repair, homeostasis, apoptosis, autophagy, regulation of metabolism, stemness, invasion, and metastasis [4,5]. Most anticancer therapeutic strategies are based on agents that activate either the p53 protein or other members of its family. However, more than 50% of cancers involve a deletion, mutation, or deficiency in the *TP53* gene. Furthermore, *p53* mutations interfere with the proper functioning of other *p53* family members, drastically diminishing the effectiveness of current treatments [6,7,8,9].

Most *p53* mutations are missense point mutations in the DNA-binding core domain, distorting its proper folding and disrupting the DNA binding and transcriptional transactivation of *p53* target genes [10]. Moreover, mutations in the *TP53* gene mainly result in the accumulation of mutant protein, leading to a loss of its tumor suppressor functions and the acquisition of novel oncogenic functions, such as increased metastasis, tumor progression, and chemoresistance [10,11,12,13,14,15,16,17,18].

These mutations exhibit several features. For example, mutations in some p53 proteins can alter the presence of the zinc ion that stabilizes the DBD of p53, thereby removing its ability to bind to target genes [19]. Furthermore, it has recently been hypothesized that the oligomerization status of mutant *p53* is important for its GOF, and that it can aggregate from simple tetramers to structures such as amyloid fibrils, although this type of aggregation seems to be independent of the OD (oligomerization domain) and appears to depend on the DBD mutation status of *p53*. Moreover, this kind of aggregation has been reported in cancer cells presenting GOF [20,21].

Various structural studies have demonstrated that mutant *p53* core domain unfolding is not irreversible, and reactivation of mutant *p53* may restore its tumor suppressor pathways, resulting in enhanced responses to current therapies. Therefore, mutant *p53* reactivation is a promising novel cancer therapy target [9]. Several strategies have been employed to restore the tumor suppressor function of mutant p53 proteins [22,23,24,25]. One of the most promising approaches is using small molecules, which are low-molecular-weight chemical entities such as metabolites, monosaccharides, lipids, and second messengers. Different mechanisms may drive the action of these molecules into reactivating tumor suppressor pathways: (1) small molecules may act as “conformation shifters” from a mutant unfolded p53 oncogenic protein toward a wild-type-like conformation, hence reactivating *p53* as a tumor suppressor transcription factor; (2) small molecules may act as destabilizers of mutant *p53* and induce its proteasomal degradation; or (3) small molecules may inhibit protein–protein interaction, allowing other members of the family, like p73, to be released from aberrant aggregation with mutant *p53* and exert its tumor suppressor functions. Consequently, various natural products, as well as a variety of drugs and xenobiotics with low molecular weight, have been tested and shown to be effective in restoring *p53* pathways [22,25,26].

Resveratrol (RES) (3,4′,5-trihydroxy-trans-stilbene), a non-flavonoid polyphenol, is a phytoalexin that is naturally found in more than 70 plant species, including peanuts, grapes, pines, and berries, and has proven antioxidant, anti-inflammatory, cardioprotective, and anticancer properties [27]. RES has been widely shown to have antiproliferative and pro-apoptotic properties, as well as the capacity to reverse multidrug resistance in various cancer types [28,29,30]. These properties appear to be enhanced by *p53* signaling pathways when tested in combination with chemotherapeutic agents [31,32]. Furthermore, it has been observed that RES’s cytotoxicity in normal cells is minimal compared with that in cancer cells, including breast and bone cells [33,34,35,36]. Additionally, it has recently been shown that RES interacts directly with other molecules and alters its bioactivity in different biological pathways [37,38,39]. In this regard, it has been demonstrated that RES can prevent mutant *p53* aggregation without affecting the *p53* wild-type complex [40]. Recently, it has been shown that zinc might reactivate mutant *p53*, restoring drug sensitivity. Interestingly, some studies demonstrate that RES can positively modulate intracellular zinc, facilitating zinc uptake and redistribution in cells [19,41,42,43,44]. Thus, RES might act as a small molecule that interacts with and inhibits mutant *p53*, thereby permitting the activation of genes in the *p53* pathway. This pilot study aims to test the antiproliferative and pro-apoptotic effects of RES in mutant *p53* cancer cell lines and to determine whether RES enhances sensitivity to commonly used chemotherapeutic agents.

## 2. Results

Four cancer cell lines with different *p53* status were chosen to address the hypothesis that RES might act as a small molecule that overpasses mutant *p53* GOF in cancer cell lines expressing only mutant forms of the protein.

### 2.1. Resveratrol’s Antiproliferative Effect Is Independent of p53 Status

**Figure 1:**
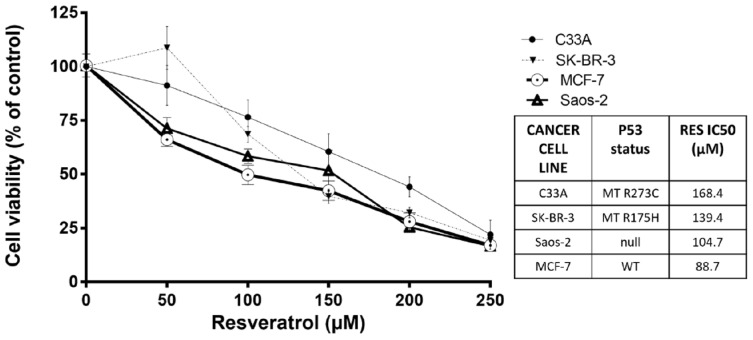
Dose-dependent effect of resveratrol (RES) on cellular metabolic activity in cancer cell lines with different *p53* status. Cells were treated with increasing concentrations of RES (50–250 µM) for 48 h. Cellular metabolic activity was assessed by means of MTT assay and normalized to that of vehicle-treated controls (EtOH 0.3% *v*/*v*). Data represent mean ± SD of at least four independent experiments. IC50 values are indicated for each cell line (presented in box).

The first step was to analyze the antiproliferative effect of RES in the selected human cancer cell lines: C33A (contact *p53* mutation in R273C), SK-BR-3 (conformational *p53* mutation in R175H), MCF-7 (*p53* wild-type), and Saos-2 (*p53* null). These cells were treated with different concentrations of RES (50, 100, 150, and 250 µM) for 48 h. After the treatment period, MTT assays were performed, and RES IC50 values were obtained. As shown in Figure 1, RES decreased cellular metabolic activity (as measured by means of MTT), consistent with reduced cell viability in all the tested cancer cell lines. As expected, RES exerted a more significant antiproliferative effect in MCF-7 cells, presenting the lowest RES IC50 value of 88.7 µM, followed by Saos-2 cells (104.7 µM), exhibiting an 18% increase in the RES IC50 value compared with the MCF-7 RES IC50 value. The mutant *p53* cancer cell lines, SK-BR-3 (139.4 µM) and C33A (168.4 µM), were significantly more resistant to the antiproliferative effect of RES, showing 57% and 90% increases in their RES IC50 values compared with the MCF-7 value, respectively (Figure 1). These results suggest that the antiproliferative effect of RES may be influenced by functional *p53* tumor suppressor pathways.

RES has a dual nature as an antioxidant in low concentrations and as a prooxidant agent in high concentrations, directly stimulating or inhibiting the activity of nicotinamide adenine dinucleotide phosphate (NADPH)-dependent cellular oxidoreductase enzymes, respectively. Given that this can be measured by means of MTT assay, we added photographs of the untreated and treated SK-BR-3 cell line after 48 h of treatment as a Supplementary Figure (Figure S1). The qualitative observations are consistent with the reduction in metabolic activity measured via MTT and support a decrease in cell number following treatment.

### 2.2. Resveratrol Induces Apoptosis in Mutant p53 Cancer Cell Lines

**Figure 2:**
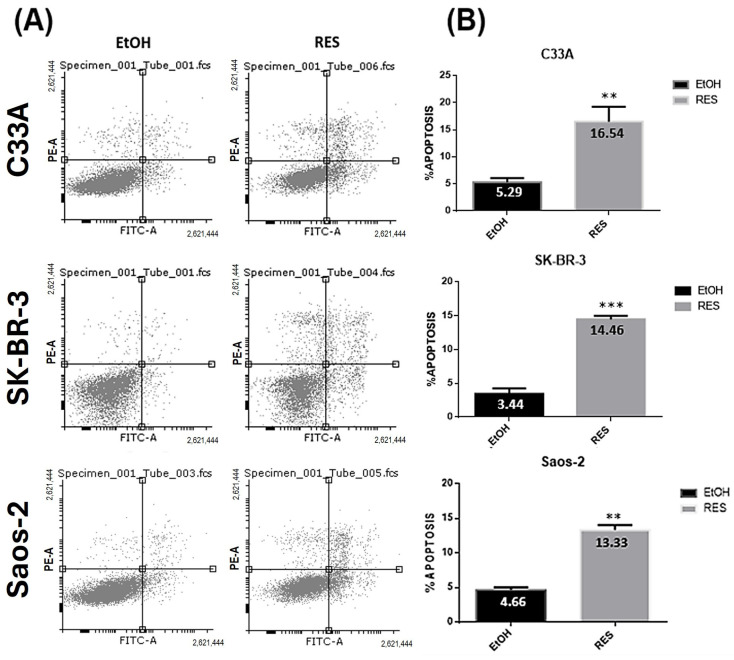
Resveratrol induces apoptosis in cancer cell lines with mutant or null *p53*. (**A**) C33A, SK-BR-3, and Saos-2 cells were treated with vehicle (ethanol) or resveratrol at their respective IC50s for 48 h. Apoptosis was assessed by means of Annexin V/propidium iodide (PI) double staining followed by flow cytometry analysis. Representative dot plots show viable cells in the lower left quadrant (Annexin V^−^/PI^−^), early apoptotic cells in the lower right quadrant (Annexin V^+^/PI^−^), and late apoptotic cells in the upper right quadrant (Annexin V^+^/PI^+^). (**B**) Quantifications of total apoptotic cells (early + late apoptosis) are shown as the mean ± SD from three independent experiments. ** *p* < 0.01; *** *p* < 0.001 versus EtOH. RES, resveratrol; EtOH, control with ethanol.

Apoptosis assays were performed to evaluate whether RES’s antiproliferative effect is related to cytotoxicity. Cancer cell lines were treated with their respective RES IC50s for 48 h. After this treatment, apoptosis assays were performed according to the protocol of the Alexa Fluor 488 Annexin V/Dead Cell Apoptosis Kit. Apoptosis levels increased by only ~15% in the mutant *p53* (C33A and SK-BR-3) cancer cell lines, whereas in *p53*-null Saos-2 cells, there was only an 8.66% increase after treatment with RES compared with the vehicle (EtOH) control (Figure 2). The figure shows that RES alone was not as efficient at driving mutant *p53* cells to apoptosis as it was with MCF-7 cells, which showed ~60% total apoptosis, as previously reported by our group [31]. These results suggest that the pro-apoptotic effect of RES is more pronounced in the presence of functional wild-type *p53*, which is consistent with the cell viability assay results.

### 2.3. Resveratrol Induces the Expression of Key Antiproliferative and Pro-Apoptotic p53 Target Genes in Mutant and Null p53 Cancer Cell Lines

**Figure 3:**
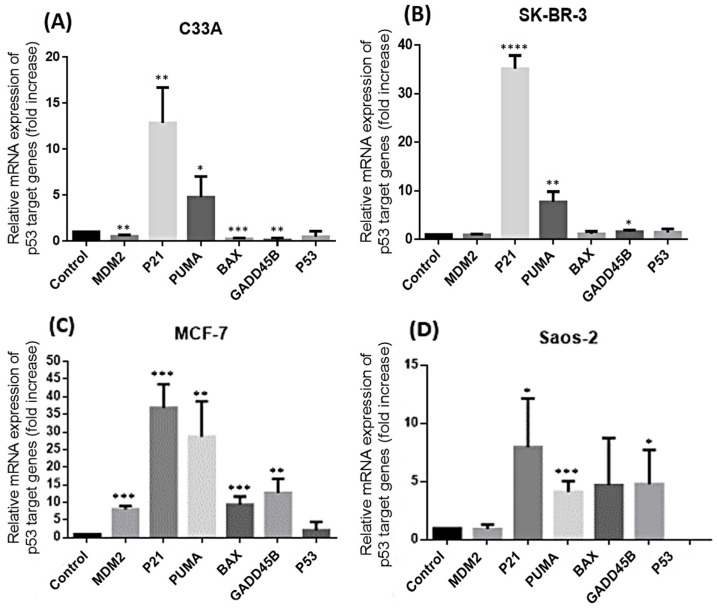
Resveratrol induces the expression of *p53* target genes across different *p53* backgrounds. Gene expression analysis was performed in (**A**) C33A (*p53* R273C), (**B**) SK-BR-3 (*p53* R175H), (**C**) MCF-7 (*p53* WT), and (**D**) Saos-2 (*p53* null) cells following treatment with vehicle (EtOH) or resveratrol (RES) at their respective IC50s for 48 h. Relative mRNA levels of *p53* target genes were determined by means of RT-qPCR and expressed as fold changes relative to vehicle-treated controls, normalized to the housekeeping gene β2-microglobulin (β2M). Data are presented as the mean ± SD of at least three independent experiments. Statistical significance was evaluated using one-way ANOVA followed by appropriate post hoc multiple comparison tests. * *p* < 0.05; ** *p* < 0.01; *** *p* < 0.001; **** *p* < 0.0001 versus control.

As the antiproliferative and pro-apoptotic effects of RES are enhanced in *p53* wild-type MCF-7 cells, we analyzed its effect on the expression of *p53* tumor suppressor target genes: *MDM2*, *P21*, *PUMA*, *BAX*, *GADD45*, and *TP53*. Each cancer cell line was treated with its respective RES IC50s for 48 h. RT-qPCR analysis was performed to identify expression fold changes in the previously mentioned genes. Consistent with the cell viability assay, MCF-7 showed the highest fold increase in every tested gene: *MDM2* (mean fold increase: 8.065), *P21* (36.85), *PUMA* (28.70), *BAX* (9.395), *GADD45* (12.68), and *TP53* (0.4731) (Figure 3C). Even though the expression of all these genes, except for *TP53*, was also observed in *p53*-null Saos-2 cells, the mean fold increase was significantly lower than that observed in MCF-7 cells for *MDM2* (0.9610), *P21* (7.982), *PUMA* (4.134), *BAX* (4.713), and *GADD45* (4.806) (Figure 3D). On the other hand, in the mutant *p53* cancer cell lines, we observed only a clear fold increase in the expression of the *P21* and *PUMA* genes, but it was still significantly lower than the changes observed in MCF-7 (C33A: *P21* (13.99), *PUMA* (4.775); SK-BR-3: *P21* (34.75), *PUMA* (7.728)) (Figure 3A and Figure 3B, respectively). As expected, RES’s anticarcinogenic effects in *p53* WT are mainly mediated by the activation of wild-type *p53*. Nevertheless, in *p53*-deficient environments, RES is still capable of regulating the expression of *p53* genes, suggesting that RES may be stimulating the expression of those genes via an alternative pathway or by liberating or inducing the activation of other *p53* family members like *p73*, a hypothesis that still needs to be elucidated.

### 2.4. Resveratrol Induces Sensitivity to Cisplatin in Mutant p53 Cancer Cell Lines

**Figure 4:**
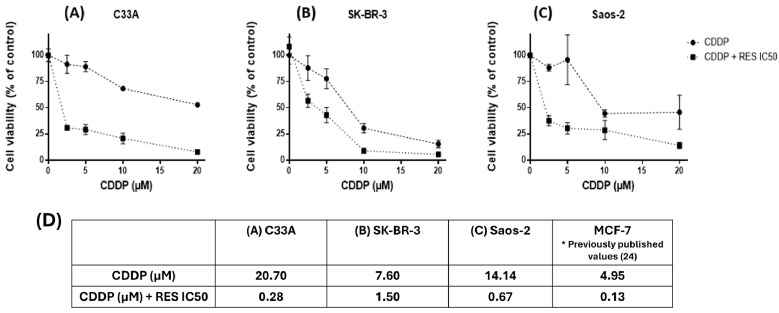
Resveratrol enhances cisplatin sensitivity in cancer cell lines with mutant or null *p53*. (**A**) C33A, (**B**) SK-BR-3, and (**C**) Saos-2 cells were treated with increasing concentrations of cisplatin (CDDP; 2.5–20 μM) in the presence or absence of resveratrol (RES), at the corresponding IC_50_ for each cell line, for 48 h. Cellular metabolic activity was assessed via MTT assay and normalized to that of vehicle-treated controls. Data represent the mean ± SD of at least four independent experiments. (**D**) IC50 values for CDDP were calculated using non-linear regression analysis (log [CDDP] vs. normalized response, variable slope model) using GraphPad Prism 6.01. * Previously published results from our working group.

As RES has previously been shown to sensitize wild-type *p53* cancer cells to CDDP (cisplatin) [31], we tested whether this effect could be observed in mutant and *p53*-null cancer cell lines. Cells were treated with different concentrations of CDDP (2.5, 5, 10, and 20 µM) alone or in combination with their respective RES IC50s for 48 h. After the treatment period, MTT assays were performed. The IC50 values for CDDP were calculated via nonlinear regression (curve fit) of log [CDDP] vs. the normalized response–variable slope. Our results show that RES induced sensitivity to CDDP in mutant and *p53*-null cells (Figure 4). As expected, the mutant *p53* cancer cell lines C33A and SK-BR-3 were more resistant to CDDP treatment than the *p53* wild-type MCF-7 cancer cell line. Interestingly, Saos-2 (null *p53*) exhibited high resistance to CDDP treatment as well (Figure 4D). Even though the grade of resistance differed across the tested cell lines, the effect of RES in sensitizing cancer cells to CDDP was significant and consistent regardless of *p53* status. Thus, the specific mechanisms through which RES drives the sensitization of mutant and *p53*-null cells remain to be elucidated.

## 3. Discussion

Cancer caused 9.9 million deaths in 2020, with the annual toll estimated to rise to 16.4 million by 2040 [1]. Ninety percent of deaths in cancer patients receiving traditional chemotherapeutics or novel targeted drugs are related to various multidrug resistance (MDR) mechanisms such as elevated enhanced drug efflux, xenobiotic metabolism, increased DNA repair capacity, genetic factors (gene mutations, amplifications), and epigenetic alterations [45]. Due to its critical tumor suppressor role in preventing carcinogenesis, the TP53 gene is often found to have mutations in human cancers. These mutations are commonly associated with a poor prognosis and a more malignant and resistant cancer phenotype. TP53 gene mutations often result in changes in protein conformation and the mutant protein’s overexpression and aberrant accumulation, drastically reducing or abolishing its tumor suppressor function. This situation has been widely associated with resistance to standard medications, including cisplatin, anthracyclines (doxorubicin), alkylating agents (temozolomide), antiestrogens (tamoxifen), EGFR inhibitors (cetuximab), and antimetabolites (gemcitabine) [46].

On the other hand, phytochemicals have strongly attracted the attention of researchers. Many phytochemicals exert a broad range of protective benefits, from reducing inflammation and speeding healing to preventing infection and fighting cancer. RES is considered a promising compound because of its anti-inflammatory, antioxidant, and cancer-prevention effects, along with its differential effects on cancer and normal cells. RES has been shown to regulate cancer signaling pathways, including SIRT1, p53, p21, AMPK, ROS, BMP7, COX-2, NO, caspases, Wnt, TNFs, NF-κB, EMT, and the pentose phosphate pathway [47].

In this study, RES demonstrated an antiproliferative effect on cancer cells regardless of their *p53* status. Our results are consistent with previously published findings indicating that RES’s anticarcinogenic effects are primarily driven by *p53* tumor suppressor mechanisms. Accordingly, MCF-7 cells expressing wild-type *p53* are more sensitive to RES treatments, as reflected in the lowest obtained RES IC50 value (RES IC50 88.7 µM). *p53*-null Saos-2 cells were slightly more resistant (RES IC50 104.7 µM), while mutant *p53* SK-BR-3 and C33A were drastically more resistant to RES (RES IC50 139.4 and 168.4 µM, respectively). These results are also consistent with the literature, which states that the loss of *p53* expression or function is a driver of unregulated cell growth. Hence, the greater the cell division rate, the more rapidly a tumor cell gains the capacity to acquire multiple mutations. Moreover, the expression of mutant forms of the *p53* protein is commonly associated with oncogenic gain of function, which often results in enhanced proliferation and evasion of cell cycle arrest and apoptosis.

The main aim of using RES is to improve the efficiency of current therapies in driving cancer cells to death. Our group previously published results showing that RES induces *p53* phosphorylation at Serine 20, which is required for the activation of *p53* target genes. In addition, RES induces sensitivity to CDDP in normal and cisplatin-resistant MCF-7 cells, as evidenced by a drastic decrease in their CDDP IC50 when treated with RES at 100 µM [31]. In this study, we showed RES’s capacity to induce apoptosis in mutant *p53* SK-BR-3 and C33A (averaging around 15%) and in *p53*-null Saos-2 cells (averaging around 13%), although all apoptosis rates were lower than those reached in MCF-7 *p53* wild-type cells (average around 60%) in the previously mentioned study.

To connect the role of *p53* with RES’s antiproliferative and pro-apoptotic effects, we further analyzed the expression of the most common *p53* tumor suppressor target genes in mutant *p53* cancer cell lines treated with their RES IC50s for 48 h. We observed a small increase in *P21* and *PUMA* gene expression in C33A and SK-BR-3, despite *p53* mutations in these cell lines. This is interesting because Huang Y. previously reported that mutant *p53* drives cancer chemoresistance due to a loss of function in activating *PUMA* transcription, a situation that was overcome by inducing the expression of wild-type *p53* in mutant *p53* colorectal cancer cells and rescuing subsequent *PUMA*-induced apoptosis [48]. It is known that the analyzed *p53* target genes’ expression is not only regulated by *p53* due to its cellular importance; thus, even though the expression of more *p53* target genes (*MDM2*, *P21*, *PUMA*, and *GADD45*) was increased in *p53*-null Saos-2, the expression of these genes was also significantly greater in *p53* wild-type MCF-7 cells, confirming once again that RES induces the expression of tumor suppressor genes mediated mostly by the action of *p53* [31,48]. Furthermore, the increased expression of *p53* target genes in *p53*-null Saos-2 cells compared with *p53* mutant cells, which only exhibited significant increases in *P21* and *PUMA*, did not lead to increased apoptosis levels in Saos-2 cells over mutant cancer cell lines as we expected. The Saos-2 apoptosis levels were lower than those observed in the mutant *p53* cancer cell lines C33A and SK-BR-3. This could be related to differences in apoptosis inhibition regulation between the Saos-2 *p53*-null cell line and the *p53* mutant cell lines C33A and SK-BR-3, in which the presence of the *p53* mutant forms could be the main factor blocking *p53*-independent or -dependent apoptosis. Although our findings are consistent with the hypothesis that RES exerts a greater effect in cells expressing either wild-type or mutant *p53*, we note an important limitation. Specifically, using cell line-specific IC50s enabled normalization of cytotoxic stress within each model, but it precluded direct quantitative comparisons across cell lines. Because each cell line was treated with a different effective resveratrol dose, the observed differences in apoptosis and gene expression responses in wild-type, mutant, and null *p53* contexts should be interpreted with caution. Future studies that apply matched, common-dose conditions across cell lines would help to better clarify these comparative effects.

Current novel therapies rely on natural compounds that might enhance the effects of common chemotherapeutic strategies, and RES has long been considered in this context. Our laboratory previously showed that in MCF-7 cells driven to a CDDP-resistant phenotype (MCF-7R), RES can drastically sensitize both MCF-7R and MCF-7 normal cells to CDDP treatment through *p53*-dependent pathways [31]. In our study, we showed that this capacity of RES extends to mutant *p53* cancer cell lines. When such a cell line is treated with CDDP in combination with RES at its RES IC50, the CDDP IC50 drastically diminishes. These results lead us to question how RES achieves its chemosensitization effect in a *p53*-deficient context and to determine whether RES acts as a reactivator of mutant *p53* toward its tumor suppressor functions. Although the reduction in the cisplatin IC50 observed in combination with resveratrol was substantial, the present study was not designed to formally assess drug synergy using quantitative models such as Chou–Talalay or Bliss independence. Therefore, the observed effects are interpreted as sensitization rather than a demonstrated synergistic interaction. Future studies incorporating combination matrices and formal synergy analyses will be necessary to define the nature and strength of this interaction.

It has been shown that some *p53* mutants form aggregates that are important in cancer progression and can also induce the aggregation of p73 [49]; thus, the disaggregation of these complexes should have an antitumor effect in cancer cells. Accordingly, it has been previously demonstrated that RES directly interacts with the core domain of *p53* and abrogates the aberrant accumulation of mutant forms of the protein, as well as other forms of amyloid aggregation [40,50]. Importantly, the present study did not directly assess the mutant *p53* conformation or aggregation state; therefore, the proposed aggregation–disruption model should be interpreted as a plausible but untested mechanism in this experimental context. Given that in our study, cells with *p53* wild-type status showed much more efficient inhibition of proliferation and induction of apoptosis than cells with mutant *p53* status, we hypothesize that RES’s reactivation of mutant *p53* is limited. Although RES producing a change in conformation has not been described in the literature, we cannot discard other minor but important changes, including key phosphorylation sites such as ser 20 that may reactivate apoptosis capacity. Instead, we speculate that the alleged direct interaction of RES with *p53* might change the stability and aggregation of the mutant *p53* protein, thus potentially allowing other members of its family (such as p73 or p63) to be released and perform tumor suppressor functions; however, this remains speculative and was not directly evaluated in this study [51]. This could be reflected in RES’s induction of the *p53* target genes *P21* and *PUMA* in the *p53* mutant cell lines at a much lower expression level than in wild-type cells, as shown in other works [52]. We also speculate that RES’s capacity to positively modify zinc status in the cell may also contribute to inhibiting the formation of *p53* mutant aggregates. A limitation of the present study is the absence of protein-level analyses to directly evaluate changes in *p53*’s stability, post-translational modifications, or downstream effector proteins. While transcriptional activation of *p53* target genes suggests engagement of *p53*-related pathways, these data do not allow us to distinguish among the direct reactivation of mutant *p53*, modulation of its stability or aggregation state, or indirect activation of *p53* family members such as *p73*. Future studies incorporating protein-level analyses will be necessary to clarify these mechanisms.

In contrast, in the Saos-2 cell line, RES may activate the genes of interest through *p53*-independent mechanisms. This could involve *p53* family members such as *p73*; however, this hypothesis requires experimental validation. Additional studies are needed to elucidate the mechanisms by which RES sensitizes mutant *p53* cancer cell lines to current chemotherapeutic agents. Overall, these findings further support the promising potential of RES to improve existing therapies.

## 4. Materials and Methods

### 4.1. Reagents

Resveratrol (RES), cisplatin (CDDP), and 3-(4,5-dimethylthiazol-2-yl)-2,5-diphenyltetrazolium bromide (MTT) were purchased from Sigma-Aldrich (St. Louis, MO, USA).

### 4.2. Cell Lines and Cell Culture

The human cervical cancer cell line C33A (*p53* mutation R273C) was purchased from the American Type Culture Collection (ATCC cat. no. HTB-31 [C-33A]; Manassas, VA, USA). The breast cancer cell lines SK-BR-3 (*p53* conformational mutation R175H) and MCF-7 (*p53* wild-type) and the osteosarcoma cell line Saos-2 (*p53* null) were donated by Dr. Alfredo Lagunas Martínez (National Institute of Public Health, Center for Research on Infectious Diseases; Mexico City).

All cell lines were cultured in Dulbecco’s modified Eagle’s medium (DMEM) supplemented with 10% (*v*/*v*) fetal bovine serum, penicillin (100 U/mL), and streptomycin (100 μg/mL), incubated at 37 °C in a humidified 5% CO_2_ atmosphere. RES and CDDP stock solutions were prepared at 80 mM in absolute ethanol (EtOH) and DMSO, respectively. Both compounds were diluted in supplemented culture medium at the final concentration indicated in each experiment.

### 4.3. Cell Viability Assays

Briefly, 2.5 × 10^4^ cells were seeded in 24-well plates 48 h before treatment. All cell lines were treated with different concentrations of RES (50, 100, 150, and 250 µM), CDDP (1, 2.5, 5, 10, and 20 µM), or CDDP (1, 2.5, 5, 10, and 20 µM) in combination with RES IC50 for 48 h. After the incubation period, mitochondrial activity was measured using the modified 3-[4,5-dimethylthiazol 2-yl]-2,5 diphenyltetrazolium bromide (MTT) assay, which reflects cellular metabolic activity and is commonly used as a proxy for cell viability [38]. Cells were incubated with MTT (0.5 mg/mL) for 30 min at 37 °C. The medium was removed, precipitated formazan crystals were solubilized with 500 µL of acid isopropanol, and absorbance was measured at 570 nm (Tecan’s Sunrise absorbance microplate reader, Tecan Group Ltd., Männedorf, Switzerland). The growth percentage was calculated using the number of control cells with the vehicle as 100% at 48 h.

### 4.4. Apoptosis Assay

Cells were plated at a density of 2.5 × 10^5^ cells/dish in p60 cell culture dishes 48 h before the treatment. Apoptosis analysis was performed using the Alexa Fluor 488 Annexin V/Dead Cell Apoptosis Kit (Invitrogen V13245; CA, USA). Briefly, the culture medium was recovered, and cells were harvested. After centrifugation, cells were washed with cold PBS 1× and washed once more with Annexin binding buffer 5× (ABB). After centrifugation, cells were softly resuspended in 100 µL incubation mix (94 µL AAB 1×, 5 µL Alexa Fluor 488 Annexin, and 1 µL propidium iodide 1× (PI)). Cells were then incubated in the dark at room temperature for 45 min. Lastly, cells were resuspended in 400 µL of ABB 1× and analyzed via flow cytometry at 530 nm and 575 nm in a FACSCalibur instrument. Data analysis was performed on 20,000 events with Summit Software Version 4.3 (Beckman Coulter Inc., Fullerton, CA, USA). Flow cytometry data were analyzed by first excluding debris based on forward scatter (FSC) and side scatter (SSC) parameters, followed by the selection of singlet cells. Quadrants were defined based on Annexin V and propidium iodide staining to distinguish viable (Annexin V^−^/PI^−^), early apoptotic (Annexin V^+^/PI^−^), and late apoptotic (Annexin V^+^/PI^+^) populations. Untreated and vehicle-treated cells were used as controls to establish gating parameters.

### 4.5. RT-qPCR

Cells were plated at a density of 2 × 10^5^ cells/dish in p60 cell culture dishes 48 h before the RES IC50 treatment. After the appropriate treatment, total RNA was extracted using TRIzol reagent (Invitrogen Life Technologies; Carlsbad, CA, USA) as described elsewhere. RNA was quantified using a NanoDrop instrument (Thermo Scientific NanoDrop One/One, Waltham, MA, USA), and an agarose gel analysis was performed to confirm integrity. Reverse transcription of total RNA was carried out using the First Strand cDNA Synthesis Kit (Thermo Fisher Scientific, Somerset, NJ, USA). Real-time RT-qPCR was performed using SYBR Green master mix (Thermo Fisher Scientific, Somerset, NJ, USA) on a 7300 Real Time PCR System instrument (Applied Biosystems, Foster City, CA, USA). The melting temperature profiles of the final products examined the specificity of each PCR. Reactions were conducted in triplicate. Relative amounts of the analyzed wild-type *p53* target genes were normalized to that of the housekeeping gene Beta-2 microglobulin (B2M). The relative changes in *p53* target gene expression data were analyzed using Livak’s 2(-Delta Delta C(T)) method [53]. Primers for *MDM2*, *P21*, *PUMA*, *BAX*, *GADD45B*, *TP53*, and *β2M* (Table 1) were purchased from Integrated DNA Technologies (IDT, Skokie, IL, USA).

### 4.6. Statistical Analysis

All results are expressed as the mean ± SD of at least three independent experiments. Dose–response curves and IC50 values for RES and CDDP were determined using non-linear regression analysis (log[drug] vs. normalized response, variable slope model) performed in GraphPad Prism 6.01. For comparisons involving multiple groups or conditions (e.g., gene expression analysis and dose–response experiments), statistical significance was evaluated using one-way or two-way ANOVA, as appropriate, followed by a multiple comparison Tukey test. For comparisons between two groups (e.g., treated vs. control conditions in apoptosis assays), statistical significance was evaluated using an unpaired two-tailed Student’s *t*-test. A *p*-value of <0.05 was considered to indicate statistical significance.

## 5. Conclusions

In summary, RES decreases cell viability, induces apoptosis, and upregulates the expression of *p53* tumor suppressor target genes, independently of *p53* status. Moreover, RES sensitizes mutant *p53* cancer cells to the chemotherapeutic agent cisplatin. Therefore, we can hypothesize that RES behaves as a small molecule that may modulate mutant *p53* function or interfere with its gain-of-function (GOF) activities, potentially relieving mutant *p53*-mediated inhibition of *p53*-family signaling pathways, rather than acting as a direct reactivator of wild-type *p53* activity. However, these mechanisms remain speculative and require further experimental validation. The current preliminary results are consistent and are a first step toward elucidating the effect of RES on mutant *p53* cancer cell lines. Conclusions should be softened, and this work is framed explicitly as a phenotypic study consistent with, but not direct proof of, the aggregation–disruption model.

## Figures and Tables

**Table 1:** Sequences of primers used for reverse-transcription quantitative PCR.

Gene	Forward (5′-3′)	Reverse (5′-3′)
*MDM2*	AGAAGGACAAGAACTCTCAGATG	GTGCATTTCCAATAGTCAGCTAA
*P21*	GCAGACCAGCATGACAGAT	GAGACTAAGGCAGAAGATGTAGAG
*PUMA*	CACCTAATTGGGCTCCATCT	ACGACCTCAACGCACAGTA
*BAX*	CAAACTGGTGCTCAAGGC	AAAGATGGTCACGGTCCAAC
*GADD45B*	GGGAAGGTTTTGGGCTCTCT	GGTCACCGTCTGCATCTTCTG
*TP53*	GACACGCTTCCCTGGATTG	GACGCTAGGATCTGACTGC
*β2M*	GGACTGGTCTTTCTATCTCTTGT	ACCTCCATGATGCTGCTTAC

## Data Availability

The data presented in this study are available on request from the corresponding authors.

## Supplementary Materials

The following supporting information can be downloaded at: https://www.mdpi.com/article/10.3390/ijms27104481/s1.

## Abbreviations

The following abbreviations are used in this manuscript:

*TP53*	Tumor suppressor gene that produces the p53 protein
DNA	Deoxyribonucleic acid
GOF	Gain-of-function
DBD	DNA-binding domain
IC50	Half-maximal inhibitory concentration
RES	Resveratrol
MTT	A colorimetric assay used to measure the metabolic activity of living cells, which is proportional to their viability
RT-qPCR	Reverse transcription and quantitative real-time PCR
SD	Standard deviation
CDDP	Cisplatin
OD	Oligomerization domain
