# Supplementary material for: Reactivation of P53 Antiproliferative and Pro-Apoptotic Pathways by Resveratrol in Mutant P53 Cancer Cell Lines
Source: Int J Mol Sci. 2026 May 16;27(10):4481. doi: 10.3390/ijms27104481 (PMC13207096; doi:10.3390/ijms27104481)
Supplement: Supplementary file 1 [file ijms-27-04481-s001.zip › ijms-4300435-supplementary.pdf]

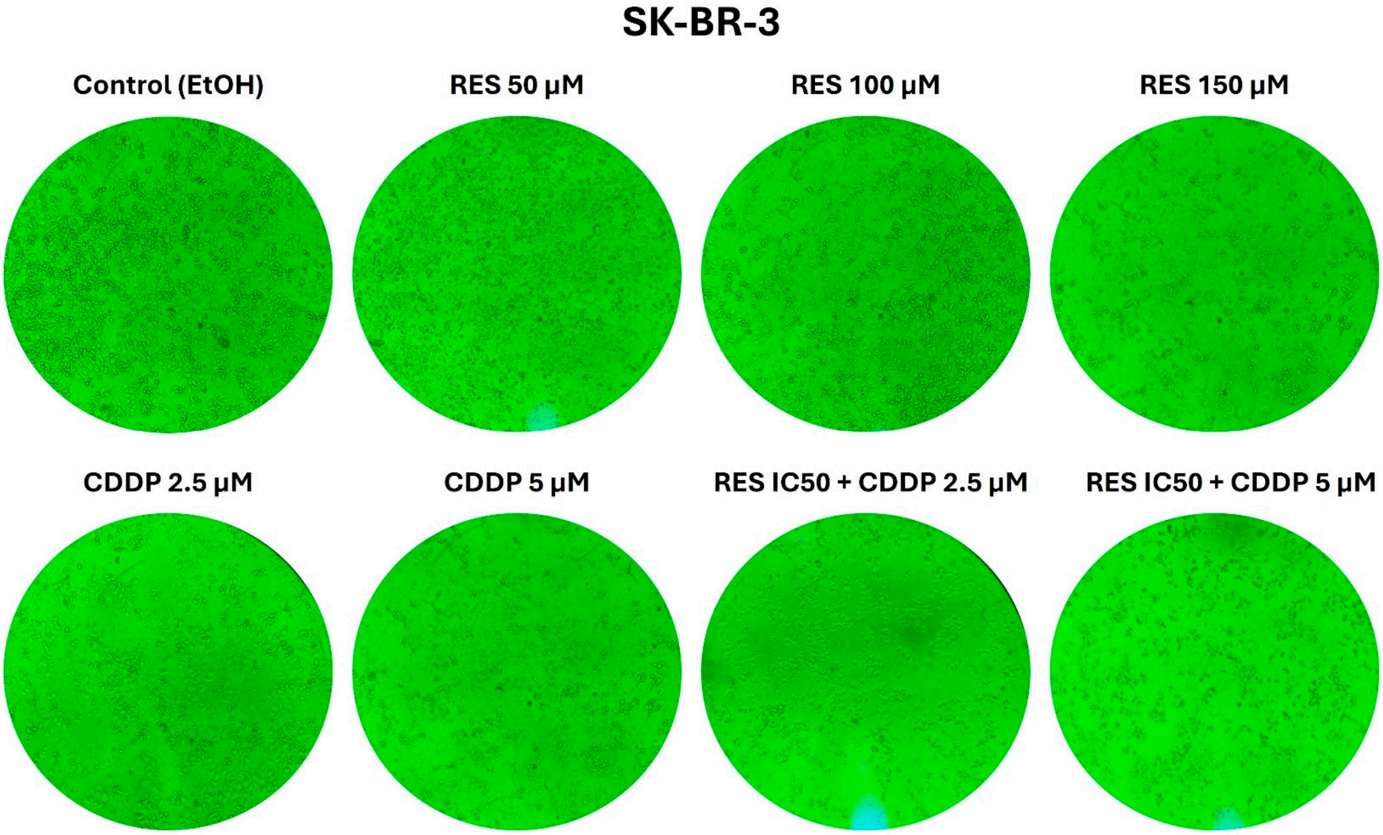

Figure S1. Representative images of SK-BR-3 cells following treatment with resveratrol (RES) and cisplatin (CDDP). Cells were left untreated or treated with RES and/or CDDP for 48 h. Representative bright-field images show a progressive reduction in cell density and morphological changes consistent with decreased cell number at higher RES concentrations. Images were acquired using a green-contrast visible-light optical microscope equipped with a 48-megapixel camera. EtOH: ethanol (vehicle control); IC50: half-maximal inhibitory concentration.
